# A Real-World Safety Profile in Neurological, Skin, and Sexual Disorders of Anti-Seizure Medications Using the Pharmacovigilance Database of the Korea Adverse Event Reporting System (KAERS)

**DOI:** 10.3390/jcm13133983

**Published:** 2024-07-08

**Authors:** Dajeong Kim, Sukhyang Lee

**Affiliations:** 1 Department of Biohealth Regulatory Science, College of Pharmacy, Ajou University, Suwon 16499, Republic of Korea; 2 Division of Clinical Pharmacy, College of Pharmacy, Ajou University, Suwon 16499, Republic of Korea

**Keywords:** adverse event reporting system, anti-seizure medication, adverse reproductive outcome, sodium channel blockers, epilepsy, Korea Adverse Event Reporting System Database (KAERS DB), pharmacovigilance

## Abstract

(1) **Background**: The utilization of high-quality evidence regarding the safety of anti-seizure medications (ASMs) is constrained by the absence of standardized reporting. This study aims to examine the safety profile of ASMs using real-world data. (2) **Methods**: The data were collected from the Korea Adverse Event Reporting System Database (KAERS-DB) between 2012 and 2021. In total, 46,963 adverse drug reaction (ADR)–drug pairs were analyzed. (3) **Results**: At the system organ class level, the most frequently reported classes for sodium channel blockers (SCBs) were skin (37.9%), neurological (16.7%), and psychiatric disorders (9.7%). For non-SCBs, these were neurological (31.2%), gastrointestinal (22.0%), and psychiatric disorders (18.2%). The most common ADRs induced by SCBs were rash (17.8%), pruritus (8.2%), and dizziness (6.7%). Non-SCBs were associated with dizziness (23.7%), somnolence (13.0%), and nausea (6.3%). Rash, pruritus, and urticaria occurred, on average, two days later with SCBs compared to non-SCBs. Sexual/reproductive disorders were reported at a frequency of 0.23%. SCBs were reported as the cause more frequently than non-SCBs (59.8% vs. 40.2%, Fisher’s exact test, *p* < 0.0001). (4) **Conclusions**: Based on real-world data, the safety profiles of ASMs were identified. The ADRs induced by SCBs exhibited different patterns when compared to those induced by non-SCBs.

## 1. Introduction

Epilepsy is the third most common neurological disorder, following stroke and dementia [1,2]. It affects approximately 50 million people worldwide, with a lifetime prevalence of 7.6 per 1000 persons. In Korea, the incidence and prevalence of epilepsy were 35.4 per 100,000 persons and 4.8 per 1000 persons, respectively, in 2017 [3,4]. Epilepsy affects individuals across all age groups, with a higher incidence observed in children compared to youth and middle-aged individuals, and an even more pronounced prevalence in the elderly. The incidence curve exhibits a U-shape, which significantly increases and transitions to a J-shape after the age of 60 [2,4,5].

The pharmacotherapy of epilepsy typically begins with monotherapy. It is expected that 70% of all patients with epilepsy will achieve remission through the use of the appropriate anti-seizure medications (ASMs) [6]. The mechanisms of ASMs are classified into modulation of voltage-dependent ion channels, potentiation of γ-amino butyric acid, multiple mechanisms of action, and another mechanism of action [7]. The choice of ASM is primarily based on the type of epilepsy. In addition, it is essential to consider the pharmacokinetic properties of the drug, potential drug interactions, the age and sex of the patient, comorbidities, and adverse events [8]. Adverse events lead to the discontinuation of ASMs in 1.35% of patients [9]. The long-term safety of ASMs is associated with chronic and cumulative effects, as well as rare but potentially serious idiopathic reactions, delayed onset of adverse effects, and other related concerns [10]. ASMs have the potential to cause central nervous system-related disorders by pathologically suppressing the overactivation of neurons. It has been reported that most ASMs may cause dose-dependent side effects, such as sedation, somnolence, incoordination, nausea, and fatigue [11,12]. Other important safety issues of ASMs include sexual and reproductive disorders, such as sexual dysfunction [13,14] and teratogenicity [15,16]. The use of ASMs during pregnancy may affect fetal cognitive and behavioral development, both in the early and full-term stages [15]. The impact of paternal exposure to ASMs on offspring is a controversial topic. Paternal valproate exposure led to behavioral alterations in mice [17]. In other studies, no correlation was founded between paternal exposure to valproate and cognitive disorders in offspring [18,19]. Epilepsy is a chronic neurological disorder that requires long-term pharmacotherapy. Therefore, it is necessary to develop individual clinical strategies that take into account the safety profile of ASMs, as the possibility of adverse events inevitably increases [20]. This study aims to analyze the patterns of ADRs based on the mechanisms of action of ASMs, with a focus on major ADRs, including neurological, dermatological, and sexual/reproductive disorders, using real-world data.

## 2. Materials and Methods

### 2.1. Study Design and Data Source

Korea Adverse Event Reporting System (KAERS) is an online system designed to facilitate the management of adverse event reports associated with post-marketing drugs [21]. The KAERS-DB is a unified and analyzable database that underwent screening and cleansing to remove input errors, logical errors, and other potential issues. This retrospective study analyzed ASM-induced ADRs using nationwide spontaneous reporting data from the KAERS-DB between 2012 and 2021. The following cases were excluded from the analysis: those with incomplete data (*n* = 900), those where ASMs were not reported as suspected drugs (*n* = 104,126), those with logical errors (*n* = 1034), and those with missing adverse event information (*n* = 1010). A total of 60,902 ADR-ASM pairs were generated. Only reports with a causality category level of possible or higher were included in the analysis, in accordance with the criteria of the World Health Organization’s Uppsala Monitoring Centre (WHO-UMC). The study design is summarized in Figure S1 in the Supplementary Appendix.

### 2.2. Identification of Anti-Seizure Medications

Study drugs were screened according to their label indications approved by the Ministry of Food and Drug Safety (MFDS). If the number of marketed products containing the same active substance is less than two, such as perampanel and brivaracetam, the KAERS-DB does not provide the relevant data for analysis due to the possible disadvantages to the marketing company under the guidelines of the institute. The selected 12 ASMs were carbamazepine, clonazepam, gabapentin, lacosamide, lamotrigine, levetiracetam, oxcarbazepine, phenobarbital, phenytoin, pregabalin, topiramate, and valproate. The drugs were classified as either sodium channel blockers (SCBs) or non-SCBs based on their mechanism of action. SCBs comprised carbamazepine, lacosamide, lamotrigine, oxcarbazepine, phenytoin, topiramate, and valproate, while non-SCBs included clonazepam, gabapentin, levetiracetam, phenobarbital, and pregabalin (Table S1).

### 2.3. Definition of Adverse Drug Reactions

An ADR is an unintended, harmful event attributed to the use of medications [22]. An adverse event was considered as an ADR only if the causality was assessed as possible, probable, or certain according to the World Health Organization's Uppsala Monitoring Centre (WHO-UMC) criteria. ADRs were coded according to the Preferred Term (PT) and System Organ Class (SOC) of the World Health Organization’s Adverse Reaction Terminology (WHO-ART). ADRs were considered serious if they resulted in death, a life-threatening situation, initial or prolonged hospitalization, disability or permanent damage, or other significant medical events. To compare the sexual and reproductive disorders caused by different drugs, the sexual/reproductive disorders were defined as any of the following conditions within the SOCs of WHO-ART: male reproductive disorders (WHO-ART code: 1410), female reproductive disorders (WHO-ART code: 1420), fetal disorders (WHO-ART code: 1500), and neonatal and infancy disorders (WHO-ART code: 1600).

### 2.4. Onset Times of Adverse Drug Reaction

The median onset time of ADRs was calculated using the date of ADR occurrence and the start date of ASM use. For the sensitivity analysis, the onset time was divided into two groups: limited to within 8 weeks due to the development time of type 2 allergic reactions (onset time 1) [23], or not limited to onset time (onset time 2).

### 2.5. Statistical Analysis

To identify ADR reporting properties, a descriptive analysis was conducted on the variables of sex, age, ADRs, drugs, and seriousness. Categorical variables were subjected to analysis using the Chi-square test or Fisher’s exact test. It was deemed to exhibit statistical significance if the *p*-value was less than 0.05.

The association between ASMs and frequently reported ADRs was estimated by calculating reporting odds ratios (RORs), proportional reporting ratios (PRRs), and information components (ICs) based on disproportionality analysis [24]. A signal was confirmed if the ADR report met all of the following criteria: the number of cases was ≥3, the ROR and PRR were ≥2, the χ^2^ ≥ 4, and the lower limit of the 95% confidence interval for IC was ≥0 [25]. All analyses were performed using SAS software, version 9.4 (SAS Institute, Cary, NC, USA), or Excel 2019 (Microsoft, Redmond, WA, USA).

## 3. Results

### 3.1. Baseline Characteristics of Adverse Drug Reactions (ADR) Reports (2012~2021)

Out of a total of 46,963 ADR-ASM pairs, 14,847 were SCBs (31.6%) and 32,116 were non-SCBs (68.4%) (Table 1). In terms of sex, 16,349 were male (34.8%), 29,454 were female (62.7%), and 1160 were unknown (2.5%). In both SCBs and non-SCBs, ADRs occurred more frequently in females than in males. Specifically, for SCBs, 8403 cases (56.6%) were reported in females compared to 5891 cases (39.7%) in males. For non-SCBs, 21,051 cases (65.5%) were reported in females compared to 10,458 cases (32.6%) in males. ADRs were more frequently reported in older individuals, particularly those aged 60 years or older (45.8%) and those in their 50 s (19.8%). In non-SCBs, individuals aged 60 years or older accounted for 55.0% of the total, indicating a more pronounced trend. The primary reporters were distributed as follows: pharmacists (39.2%), nurses (31.9%), clinicians (20.9%), customers (5.6%), and others. Out of a total of 2888 serious ADRs, 1903 cases were reported in SCBs and 985 cases were reported in non-SCBs. Serious ADRs (*n* = 2888; 6.1%) were frequently reported in association with initial or prolonged hospitalization (*n* = 1633; 3.5%), other important medical events (*n* = 1240; 2.6%), and life-threatening events (*n* = 130; 0.3%). The incidence of ADRs varied depending on the age group of the patients. Among patients under 10 years old, rash (28.6%), pruritus (9.7%), and urticaria (6.5%) were frequently reported. In patients in their 20s, rash (13.0%), dizziness (10.8%), and somnolence (7.6%) were commonly reported. The older age group experienced dizziness and somnolence more frequently. Among patients in their 50s, dizziness (19.1%) was the most commonly reported symptom, followed by somnolence (11.5%) and rash (6.4%). In patients over 60 years of age, dizziness was also the most frequently reported symptom (22.7%), followed by somnolence (10.9%) and nausea (5.4%) (Table S2). The most common ADRs reported in male patients were dizziness (14.2%), somnolence (9.8%), and rash (9.8%). Similarly, female patients reported higher incidence of dizziness (20.8%), somnolence (10.5%), and rash (6.8%) (Table S3).

### 3.2. Analysis of Reporting Odds Ratio Based on System Organ Classes

At the level of system organ class (SOC), central and peripheral nervous system disorders accounted for 26.6% of cases, followed by skin and appendages disorders at 18.0%, gastro-intestinal system disorders at 17.9%, psychiatric disorders at 15.5%, and body as a whole—general disorders at 8.1%. In SCBs, skin and appendages disorders were the most prevalent (5631, 37.9%), while central and peripheral nervous system disorders were dominant in non-SCBs (10,007, 31.2%) (Table 2). The major drugs associated with skin and appendage disorders in SCBs were carbamazepine, lamotrigine, oxcarbazepine, phenytoin, and valproate (carbamazepine ROR 4.22, 95% CI = 3.91–4.54; lamotrigine ROR 10.96, 95% CI= 10.04–11.97; oxcarbazepine ROR 6.44, 95% CI = 5.79–7.16; phenytoin ROR 3.41, 95% CI = 2.97–3.91; valproate ROR 1.74, 95% CI = 1.61–1.87; lacosamide ROR 0.89, 95% CI = 0.7–1.13; topiramate ROR 0.52, 95% CI = 0.45–0.59) (Table 3). In non-SCBs, gabapentin and pregabalin were reported frequently for central and peripheral nervous system disorders, while clonazepam, levetiracetam, and phenobarbital were reported less frequently (gabapentin ROR 1.24, 95% CI = 1.18–1.29; pregabalin ROR 2.37, 95% CI = 2.27–2.48; clonazepam ROR 0.77, 95% CI = 0.7–0.85; levetiracetam ROR 0.37, 95% CI = 0.33–0.41; phenobarbital ROR 0.15, 95% CI = 0.08–0.26). The signal strength of psychiatric disorders was found to be significantly higher in clonazepam (ROR 3.05), followed by topiramate (ROR 1.46) and gabapentin (ROR 1.38). At the preferred term (PT) level, levetiracetam exhibited a strong correlation with an increased risk of reporting an aggressive reaction (ROR 19.1), personality disorder (ROR 13.6), psychosis (ROR 13.5), and euphoria (ROR 10.2). In the case of topiramate, depression aggravated (ROR 28.4), concentration impaired (ROR 23.6), emotional lability (ROR 9.9), and amnesia (ROR 9.3) were observed (Table S6).

### 3.3. Types of Anti-Seizure Medication-Related Adverse Drug Reactions by Drug Mechanisms

#### 3.3.1. The 20 Most Commonly Reported Adverse Drug Reactions

At the PT level, the top five ADRs observed in SCBs were rash (17.8%), pruritus (8.2%), dizziness (6.7%), urticaria (6.2%), and somnolence (3.9%). In non-SCBs, the top five ADRs were dizziness (23.7%), somnolence (13.0%), nausea (6.3%), constipation (3.7%), and vomiting (3.6%) (Table 4).

#### 3.3.2. Onset Time of Adverse Drug Reactions

The median onset time of the top 10 ADRs was compared. The median onset time for dizziness, somnolence, nausea, vomiting, constipation, mouth dryness, and dyspepsia induced by both SCBs and non-SCBs was 0 days. There was a difference in the median onset time for rash, pruritus, and urticaria between SCBs and non-SCBs (Table 5). A sensitivity analysis was performed with criteria of onset time. When the onset time was restricted to 8 weeks (onset 1), rash, pruritus, and urticaria induced by SCBs exhibited a delayed onset of 2 days compared to non-SCBs. When onset time was not restricted (onset 2), the time difference in occurrence of the rash, pruritus, and urticaria between SCBs and non-SCBs increased by 6 days, 6 days, and 7 days, respectively.

### 3.4. Sexual/Reproductive-Related Adverse Drug Reactions

With regard to sexual/reproductive ADRs (107 cases, 100%), 64 cases (59.8%) were reported in SCBs and 43 cases (40.2%) were reported in non-SCBs, showing a statistically significant difference (Fisher’s exact test, *p* < 0.0001) (Table 6).

In the SCB group, there were 44 cases of female reproductive disorders and seven cases of male reproductive disorders. The non-SCB group had 28 cases of female reproductive disorders and 15 cases of male reproductive disorders. Neonatal and infancy disorders were reported in two cases in the SCB group, while no cases were reported in the non-SCB group. As a result of signal detection, amenorrhea and menstrual disorder were identified for valproate, and menstrual disorder was identified for topiramate (Table S4).

## 4. Discussion

Pharmacotherapy has been demonstrated to be an effective intervention for epilepsy. 63.7% of patients who were newly diagnosed with epilepsy achieved seizure freedom within one year of receiving ASM monotherapy [26]. A total of 88% of patients taking ASMs experience one or more adverse events. Adverse events are the primary reason for early treatment discontinuation and a barrier to seizure control [27]. Understanding the safety profile of ASMs is crucial due to the long-term pharmacotherapy required to control symptoms in patients with epilepsy. There is a limitation in using high-quality evidence on ASM safety due to the lack of standardized reporting [20]. The objective of this study was to identify the primary safety concerns associated with ASMs in Korea, with a particular focus on neurological, skin, and sexual/reproductive disorders. A real-world spontaneous reporting database between 2012 and 2021 was analyzed. A total of 63,669 reports of adverse events associated with ASMs were identified nationwide in Korea, resulting in 46,963 ADR-ASM pairs with causality of possible or higher criteria of WHO assessment.

The prevalence of skin disorders in SCBs (5631/8438, 66.7%) was higher than in non-SCBs. Specifically, carbamazepine (ROR 4.22), lamotrigine (ROR 10.96), oxcarbazepine (ROR 6.44), phenytoin (ROR 3.41), and valproate (ROR 1.74) were identified as major doubtful drugs in SCBs. Topiramate (ROR 0.52) had a significantly lower incidence of skin disorders. Skin disorders were statistically less likely to occur with clonazepam (ROR 0.38), gabapentin (ROR 0.25), and pregabalin (ROR 0.19) compared to phenobarbital (ROR 4.6) in non-SCBs. ASMs are classified as the primary cause of severe cutaneous adverse reactions (SCARs) [28]. Based on data from the FDA Adverse Event Reporting System (FAERS) between 2004 and 2021, ASMs belonged to the major drug classes that caused Stevens–Johnson syndrome (SJS)/toxic epidermal necrolysis (TEN), and 19.37% of all reports were related to ASMs. Specifically, phenytoin was identified as the most frequently reported drug [29]. The study analyzing 2942 cases of drug eruption from KAERS-DB between 2008 to 2017 found that lamotrigine, valproate, carbamazepine, oxcarbazepine, levetiracetam, and phenytoin were the cause of the eruptions [30]. In this study, it was determined that carbamazepine, oxcarbazepine, lamotrigine, phenytoin, and phenobarbital were the major causative agents of skin disorders (Table S5).

On the other hand, no statistically significant ROR index was observed for lacosamide in skin disorders (ROR 0.89; 95% CI = 0.7–1.13). The effect of lacosamide on skin disorders remains a subject of debate [31,32]. Among the skin disorders, rash erythematous was detected as a signal for lacosamide (Table S5). Drug hypersensitivity typically occurs between 1 and 8 weeks after exposure to the drug. As most reactions occur within the first two months of treatment initiation, there is a possibility of underestimating the true incidence of the syndrome [33]. In this study, the onset of skin disorders, such as rash, pruritus, and urticaria, was delayed by 2 days in patients treated with SCBs compared to non-SCBs. Somnolence, nausea, mouth dryness, and dyspepsia occurred instantly regardless of mechanisms (Table 3). It is crucial to monitor patients treated with SCBs for delayed idiopathic hypersensitivity reactions, which may occur.

Neurological disorders were more prevalent in patients treated with non-SCBs (10,007/12,484, 80.2%). Gabapentin (ROR 1.24; 95% CI = 1.18–1.29) and pregabalin (ROR 2.37; 95% CI = 2.27–2.48) were associated with a high risk of neurological ADRs. This finding is consistent with a previous study that reported a high frequency of somnolence with pregabalin [34]. It is important to take neurological disorders seriously, as they can increase the risk of falls in the elderly [35,36,37]. There was a study that examined the relationship between ASMs and falls [38], but there is still limited information available on the specific drugs that cause them. Gabapentin and pregabalin have been identified as having a high risk of causing central and peripheral nervous system disorders. Further research is needed to determine the risk of falls associated with non-SCBs.

The estimated prevalence of psychiatric and behavioral adverse effects in adults was 8–20%, and in patients under 18 years of age, it was 11–14% [39]. The KAERS-DB reported 15.5% of ADRs with ASMs for the psychiatric disorders. Levetiracetam and topiramate are relatively well-known as causative agents [40]. The results of our analysis indicated that levetiracetam was not associated with psychiatric disorders at the SOC level. At the PT level, signal detection revealed a correlation between levetiracetam and several psychiatric ADRs, including aggressive reactions, agitation, anxiety, depression, emotional lability, euphoria, nervousness, personality disorders, psychosis, and suicide attempts. The findings of this study on topiramate were found to be consistent with those of previous studies.

Sexual/reproductive disorders (107, 100%) were more commonly reported with SCBs compared to non-SCBs (64 [59.8%] vs. 43 [40.2%], Fisher’s exact test, *p* < 0.0001). The effect on both sexes differed depending on the mechanism of action. In male reproductive disorders, including ejaculation disorder, ejaculation failure, and premature ejaculation, the number of reported cases was seven in SCBs and 15 in non-SCBs, respectively. Epilepsy can have an impact on sexual function [41], with 30% of male patients experiencing sexual dysfunction. ASMs can cause drug-induced sexual disorders. Valproate and phenobarbital have been shown to worsen sexual function, while oxcarbazepine, lamotrigine, and levetiracetam may improve it [42]. There were 44 cases of female reproductive disorders, including amenorrhea, dysmenorrhea, menorrhagia, menstrual disorder, breast discomfort, breast engorgement, breast enlargement, and breast pain in SCBs, compared to 28 cases in non-SCBs. Female patients with epilepsy who are undergoing pharmacotherapy may experience sexual dysfunction due to alternating doses of sexual hormones [43]. Valproate has been reported to induce polycystic ovary syndrome [44]. Hyperprolactinemia is known to cause amenorrhea and ejaculation disorders. A number of drugs that affect the nervous system, including phenothiazines, risperidone, selective serotonin reuptake inhibitors, monoamine oxidase inhibitors, and some tricyclic antidepressants, have been observed to induce hyperprolactinemia. There have been few studies investigating the relationship between ASMs and hyperprolactinemia. Our findings suggest that ASMs may induce typical symptoms of hyperprolactinemia.

The KAERS-DB has limitations related to non-standardized data due to reporter bias, underreporting, and heterogeneity. Incidence rates cannot be calculated due to a lack of information on the total number of patients, seizure types, indications, and comorbidities [45,46]. The KAERS-DB did not provide the data when the marketed products containing the same active substance were less than two products. Perampanel and brivaracetam were excluded for this reason. It is known that perampanel and brivaracetam are related to behavioral adverse events with irritability, anger, and aggression [47]. The third generation ASMs are currently being gradually commercialized in Korea, and further analysis will be required to assess their safety in clinical settings. Despite these limitations, this study proposed a safety profile based on real-world data from spontaneous reporting. Patients reported symptoms indicative of hyperprolactinemia associated with sexual/reproductive disorders, underscoring the importance of monitoring ASM-induced hyperprolactinemia in patients presenting with such symptoms.

## 5. Conclusions

The safety profiles of ASMs were analyzed using real-world spontaneous reporting of adverse events. SCBs had a higher likelihood of causing skin disorders, while non-SCBs had a higher likelihood of causing neurological disorders. Depending on the mechanism of ASMs, different monitoring strategies may be required, as skin disorders may occur as a delayed response when induced by SCBs. Regarding sexual/reproductive disorders, SCBs and non-SCBs had different effects on males or females.

## Figures and Tables

**Table 1:** Characteristics of reporting from the Korea Adverse Event Reporting System database (2012 to 2021).

Characteristics	Total		SCBs		Non-SCBs		*p*-Value
	N	%	N	%	N	%	
Reports	46,963	100.0	14,847	100.0	32,116	100.0	-
Sex							<0.0001
Male	16,349	34.8	5891	39.7	10,458	32.6	
Female	29,454	62.7	8403	56.6	21,051	65.5	
Unknown	1160	2.5	553	3.7	607	1.9	
Age group							<0.0001
00–09	1494	3.2	1093	7.4	401	1.3	
10–19	1580	3.4	1177	7.9	403	1.3	
20–29	2717	5.8	1775	12.0	942	2.9	
30–39	3577	7.6	1815	12.2	1762	5.5	
40–49	5319	11.3	1907	12.8	3412	10.6	
50–59	9300	19.8	2409	16.2	6891	21.5	
>60	21,509	45.8	3837	25.8	17,672	55.0	
Unknown	1467	3.1	834	5.6	633	2.0	
Original reporter							<0.0001
Clinician	9805	20.9	4495	36.0	5310	15.4	
Pharmacist	18,396	39.2	2167	17.4	16,229	47.1	
Nurse	14,974	31.9	4484	35.9	10,493	30.4	
Other medical specialists	251	0.5	167	1.3	167	0.5	
Consumer	2629	5.6	999	8.0	1630	4.7	
Unknown	908	1.9	260	2.1	648	1.9	
Assessment							<0.0001
Certain	817	1.7	399	2.7	418	1.3	
Probable/likely	11,868	25.3	5247	35.3	6621	20.6	
Possible	34,278	73.0	9201	62.0	25,077	78.1	
Seriousness							<0.0001
Yes	2888	6.1	1903	12.8	985	3.1	
No	44,075	93.9	12,944	87.2	31,131	96.9	
Seriousness category							<0.0001
Death	65	0.1	26	0.2	39	0.1	
Life-threatening	130	0.3	76	0.5	54	0.2	
Hospitalization	1633	3.5	1144	7.7	489	1.5	
Disability	31	0.1	22	0.2	9	0.0	
Congenial anomaly	2	0.0	2	0.0	-	-	
Other significant medical events	1240	2.6	818	5.5	422	1.3	

**Table 2:** Distribution of adverse drug reaction (ADR)–anti-seizure medications (ASM) pairs according to relevant System Organ Classes.

SOC	Total		SCBs		Non-SCBs	
	N	%	N	%	N	%
Total	46,963	100	14,847	100.0	32,116	100.0
Central & peripheral nervous system disorders	12,484	26.6	2477	16.7	10,007	31.2
Skin and appendages disorders	8438	18.0	5631	37.9	2807	8.7
Gastro-intestinal system disorders	8413	17.9	1351	9.1	7062	22.0
Psychiatric disorders	7290	15.5	1436	9.7	5854	18.2
Body as a whole—general disorders	3810	8.1	1078	7.3	2732	8.5
Liver and biliary system disorders	1197	2.5	662	4.5	535	1.7
Metabolic and nutritional disorders	1092	2.3	495	3.3	597	1.9
White cell and RES disorders	577	1.2	380	2.6	197	0.6
Platelet, bleeding & clotting disorders	416	0.9	308	2.1	108	0.3
Vision disorders	519	1.1	193	1.3	326	1.0
Urinary system disorders	936	2.0	146	0.98	790	2.46
Respiratory system disorders	381	0.8	130	0.9	251	0.8
Heart rate and rhythm disorders	257	0.5	101	0.7	156	0.5
Secondary terms—events	159	0.3	89	0.6	70	0.2
Musculo-skeletal system disorders	338	0.7	81	0.5	257	0.8
Cardiovascular disorders, general	212	0.5	67	0.5	145	0.5
Red blood cell disorders	87	0.2	51	0.3	36	0.1
Reproductive disorders, female	72	0.2	44	0.30	28	0.087
Hearing and vestibular disorders	69	0.1	33	0.2	36	0.1
Vascular (extracardiac) disorders	60	0.1	30	0.2	30	0.1
Special senses other, disorders	65	0.1	15	0.1	50	0.2
Neonatal and infancy disorders	11	0.0	11	0.1	-	0.0
Resistance mechanism disorders	14	0.0	8	0.1	6	0.019
Reproductive disorders, male	22	0.0	7	0.0	15	0.047
Endocrine disorders	15	0.0	6	0.0	9	0.0
Neoplasms	10	0.0	6	0.0	4	0.0
Application site disorders	8	0.0	6	0.0	2	0.0
Collagen disorders	7	0.0	3	0.0	4	0.0
Fetal disorders	2	0.0	2	0.01	-	0.000
Poison specific terms	2	0.0	-	0.00	2	0.01

SOC: system organ class; SCB: sodium channel blocker; RES: reticuloendothelial system.

**Table 3:** Signal strength of reports with anti-seizure medications at the System Organ Class level.

Group	SOC ROR (95% CI)						
Drug	Skin and Appendages Disorders	CNS & PNS System Disorders		Psychiatric Disorders		Gastro-Intestinal System Disorders	Body as a Whole—General Disorders
SCBs							
Carbamazepine	4.22 (3.91–4.54)	0.51 (0.46–0.56)		0.36 (0.31–0.41)		0.41 (0.36–0.47)	1.4 (1.24–1.57)
Lacosamide	0.89 (0.7–1.13)	1.41 (1.16–1.7)		1.09 (0.86–1.39)		0.47 (0.35–0.64)	0.34 (0.2–0.58)
Lamotrigine	10.96 (10.04–11.97)	0.16 (0.13–0.19)		0.29 (0.24–0.34)		0.21 (0.17–0.25)	1.11 (0.96–1.28)
Oxcarbazepine	6.44 (5.79–7.16)	0.36 (0.31–0.43)		0.32 (0.26–0.4)		0.29 (0.24–0.36)	0.58 (0.45–0.73)
Phenytoin	3.41 (2.97–3.91)	0.55 (0.46–0.66)		0.27 (0.2–0.37)		0.31 (0.24–0.41)	0.83 (0.64–1.09)
Valproate	1.74 (1.61–1.87)	0.48 (0.44–0.52)		0.59 (0.53–0.65)		0.64 (0.58–0.7)	0.67 (0.58–0.77)
Topiramate	0.52 (0.45–0.59)	1.21 (1.11–1.33)		1.46 (1.32–1.62)		0.56 (0.49–0.64)	0.71 (0.6–0.84)
Non-SCBs							
Clonazepam	0.38 (0.33–0.44)	0.77 (0.7–0.85)		3.05 (2.8–3.33)		0.95 (0.86–1.06)	0.84 (0.72–0.99)
Gabapentin	0.25 (0.24–0.27)	1.24 (1.18–1.29)		1.38 (1.31–1.46)		1.92 (1.83–2.02)	1.23 (1.15–1.32)
Levetiracetam	1.93 (1.78–2.09)	0.37 (0.33–0.41)		0.96 (0.86–1.06)		0.64 (0.58–0.71)	0.67 (0.58–0.78)
Phenobarbital	4.62 (3.58–5.96)	0.15 (0.08–0.26)		0.47 (0.29–0.75)		0.22 (0.12–0.4)	1.21 (0.79–1.87)
Pregabalin	0.19 (0.17–0.2)	2.37 (2.27–2.48)		1.01 (0.96–1.07)		1.47 (1.4–1.55)	1.12 (1.04–1.2)
ROR			>1		<1		Not significant

CNS: central nervous system; PNS: peripheral nervous system; SCB: sodium channel blocker; SOC: system organ classes; ROR: reporting odds ratio; CI: confidence interval.

**Table 4:** Top 20 adverse drug reactions reported to the KAERS.

Top	SCBs			Non-SCBs		
	ADR	Reports (n)	%	ADR	Reports (n)	%
1	Rash	2644	17.8	Dizziness	7597	23.7
2	Pruritus	1219	8.2	Somnolence	4184	13.0
3	Dizziness	999	6.7	Nausea	2020	6.3
4	Urticaria	916	6.2	Constipation	1193	3.7
5	Somnolence	577	3.9	Vomiting	1172	3.6
6	Nausea	359	2.4	Mouth Dry	1076	3.4
7	Hepatic Enzymes Increased	352	2.4	Rash	1003	3.1
8	Fever	333	2.2	Dyspepsia	886	2.8
9	Thrombocytopenia	285	1.9	Pruritus	849	2.6
10	Paresthesia	272	1.8	Headache	695	2.2
11	Vomiting	263	1.8	Urticaria	478	1.5
12	Headache	228	1.5	Asthenia	450	1.4
13	Leucopenia	210	1.4	Edema Generalized	442	1.4
14	Drug Hypersensitivity Syndrome	207	1.4	Hepatic Enzymes Increased	429	1.3
15	Constipation	198	1.3	Face Edema	426	1.3
16	Tremor	197	1.3	Edema	384	1.2
17	Stevens Johnson Syndrome	170	1.1	Tremor	327	1.0
18	Dyspepsia	163	1.1	Insomnia	307	1.0
19	Weight Increase	145	1.0	Weight Increase	289	0.9
20	Anorexia	139	0.9	Edema Peripheral	280	0.9
	Total of Top20	9876	66.52	Total of Top20	24,487	76.25
	Others	4971	33.5	Others	7629	23.8

SCBs: sodium channel blockers; ADR: adverse drug reaction.

**Table 5:** The median onset time of the top 10 adverse drug reactions.

	Median Time to Onset 1, within 8 Weeks Days (Q1, Q3)		Median Time to Onset 2, with No Limit Days (Q1, Q3)	
ADR	SCBs	Non-SCBs	SCBs	Non-SCBs
Dizziness	0 (0, 2)	0 (0, 1)	1 (0, 10)	0 (0, 1)
Somnolence	0 (0, 1)	0 (0, 0)	0 (0, 10)	0 (0, 1)
Rash	3 (0, 9)	1 (0, 5)	9 (1, 84)	3 (0, 72)
Nausea	0 (0, 1)	0 (0, 1)	0 (0, 6)	0 (0, 1)
Pruritus	2 (0, 7)	0 (0, 2)	7 (0, 83)	1 (0, 10)
Vomiting	0 (0, 2)	0 (0, 1)	1 (0, 6)	0 (0, 1)
Urticaria	2 (0, 8)	0 (0, 2)	8 (0, 82)	1 (0, 8)
Constipation	0 (0, 5)	0 (0, 2)	3 (0, 20)	0 (0, 5)
Mouth Dry	0 (0, 0)	0 (0, 0)	0 (0, 5)	0 (0, 0)
Dyspepsia	0 (0, 1)	0 (0, 0)	0 (0, 14)	0 (0, 0)

SCBs: sodium channel blockers; ADR: adverse drug reaction. The median onset time of ADRs was calculated using the date of ADR occurrence and the start date of ASM use. If either of these dates was missing, it was excluded from the analysis (*n* = 5684). The median time to onset 1 included results where the onset time was limited to 8 weeks (*n* = 34,069), while the median time to onset 2 included all results where the onset time was not limited (*n* = 41,252).

**Table 6:** Sexual/reproductive adverse drug reactions reported in KAERS.

Sexual/Reproductive SOC ADRs	Total	SCBs N (%)	Non-SCBs N (%)	*p*-Value SCB vs. Non-SCBs
	107 (100%)	64 (100%)	43 (100%)	<0.0001
Reproductive disorders, male	22 (20.6%)	7 (10.9%)	15 (34.9%)	0.464
Balanoposthitis	1	0	1	
Ejaculation Disorder	2	0	2	
Ejaculation Failure	3	0	3	
Ejaculation Premature	3	2	1	
Priapism	1	0	1	
Semen Abnormal	1	1	0	
Sexual Function Abnormal	11	4	7	
Reproductive disorders, female	72 (67.3%)	44 (68.6%)	28 (65.1%)	0.008
Amenorrhea	7	6	1	
Breast Discomfort	1	0	1	
Breast Engorgement	3	2	1	
Breast Enlargement	5	1	4	
Breast Pain	4	1	3	
Breast Pain Female	2	1	1	
Dysmenorrhea	3	3	0	
Gynecological-Related Pain	1	1	0	
Lactation Nonpuerperal	4	1	3	
Leukorrhea	2	1	1	
Menorrhagia	1	1	0	
Menstrual Disorder	31	23	8	
Post-Menopausal Bleeding	1	0	1	
Uterine Atony	1	0	1	
Vaginal Discomfort	1	0	1	
Vaginal Hemorrhage	2	0	2	
Vaginitis	3	3	0	
Fetal disorders	2 (1.9%)	2 (3.1%)	0 (0.0%)	-
Drug Exposure In Pregnancy	2	2	0	
Neonatal and infancy disorders	11 (10.3%)	11 (17.2%)	0 (0.0%)	-
Psychomotor Development Impaired	11	11	0	

SCB: sodium channel blockers; SOC: system organ class. The proportion of sexual/reproductive ADRs was compared between SCBs and non-SCBs using Fisher’s exact test.

## Data Availability

The data presented in this study are available on request from the corresponding author due to regulatory reasons.

## Supplementary Materials

The following supporting information can be downloaded at: https://www.mdpi.com/article/10.3390/jcm13133983/s1, Figure S1: Flow chart of constructing anti-seizure medications dataset in KAERS database; Table S1: Classification of drug groups; Table S2: The most commonly reported adverse drug reactions according to age group; Table S3: The most commonly reported adverse drug reactions according to sex; Table S4: Detected signals of adverse drug reactions in sexual/reproductive disorders associated with anti-seizure medications; Table S5: Detected signals of adverse drug reactions in skin and appendages disorders associated with anti-seizure medications; Table S6: Detected signals of adverse drug reactions in psychiatric disorders associated with anti-seizure medications.
