# Supplementary material for: A Real-World Safety Profile in Neurological, Skin, and Sexual Disorders of Anti-Seizure Medications Using the Pharmacovigilance Database of the Korea Adverse Event Reporting System (KAERS)
Source: J Clin Med. 2024 Jul 8;13(13):3983. doi: 10.3390/jcm13133983 (PMC11242241; doi:10.3390/jcm13133983)
Supplement: Supplementary file 1 [file jcm-13-03983-s001.zip › jcm-3076657-supplementary.pdf]

Figure S1. Flow chart of constructing anti-seizure medications dataset in KAERS database.

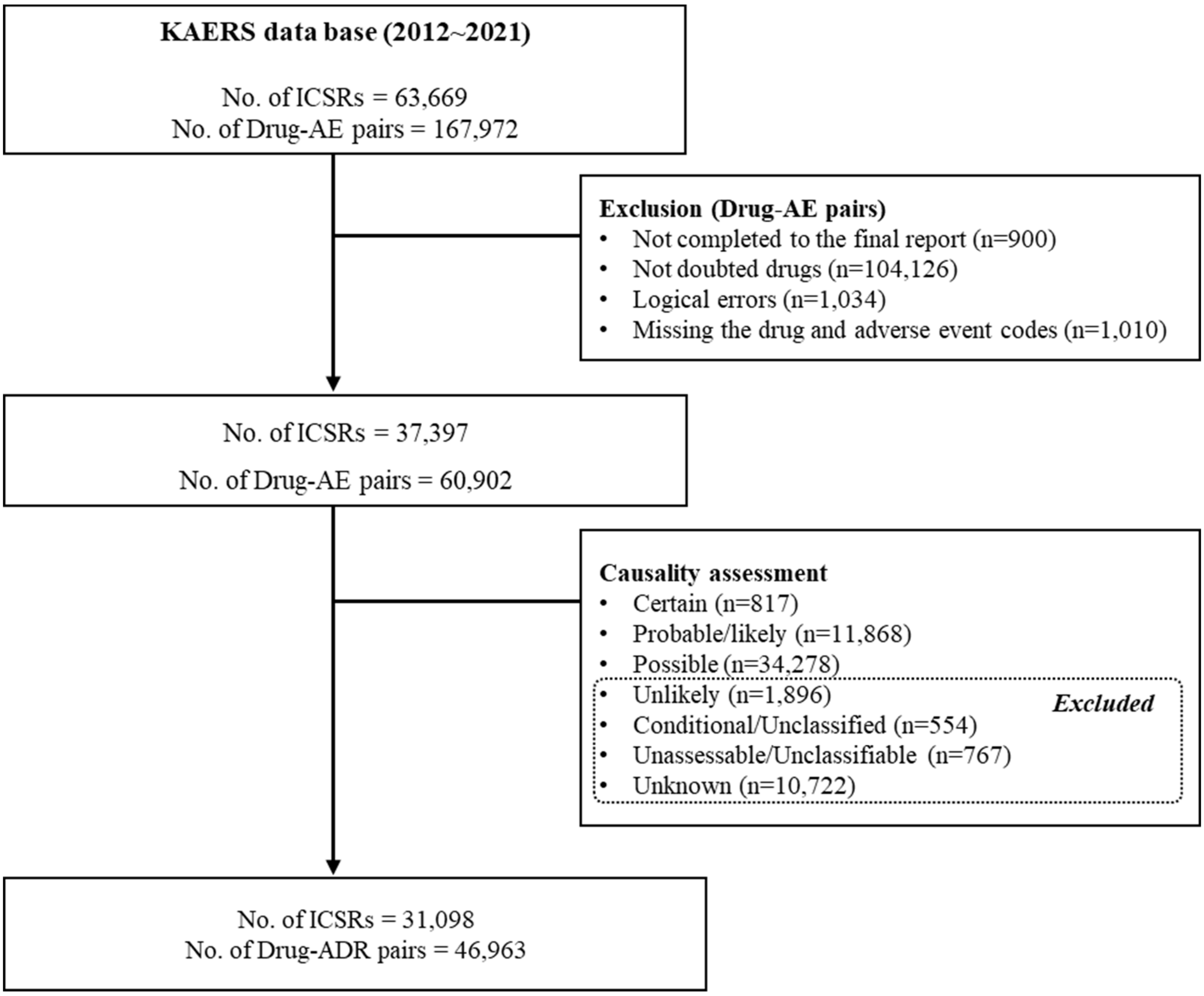

ICSR: Individual case safety report, AE: Adverse event, ADR: Adverse drug reaction

Table S1. Classification of drug groups.

Anti-Seizure medications	ATC code	Reports (*n*)	Reports (%)
SCBs		14,847	100.0
Carbamazepine	N03AF01	3,187	21.5
Lacosamide	N03AX18	478	3.2
Lamotrigine	N03AX09	2,439	16.4
Oxcarbazepine	N03AF02	1,470	9.9
Phenytoin	N03AB02	872	5.9
Topiramate	N03AX11	2,361	15.9
Valproic acid	N03AG01	4,040	27.2
Non- SCBs		32,116	100.0
Clonazepam	N03AE01	2,575	8.0
Gabapentin	N03AX12	12,704	39.6
Levetiracetam	N03AX14	3,233	10.1
Phenobarbital	N03AA02	238	0.7
Pregabalin	N03AX16	13,366	41.6

ATC code: The Anatomical Therapeutic Chemical code, SCB: Sodium channel blockers

Table S2. The most commonly reported adverse drug reactions according to age group.

ADR	< 10 years old		10s		20s		30s		40s		50s		≥ 60 years old		unknown		Total
	N	%	N	%	N	%	N	%	N	%	N	%	N	%	N	%	N
Total	1,494	100.0	1,580	100.0	2,717	100.0	3,577	100.0	5,319	100.0	9,300	100.0	21,509	100.0	1,467	100.0	46,963
Dizziness	33	2.2	81	5.1	294	10.8	498	13.9	871	16.4	1,778	19.1	4,886	22.7	155	10.6	8,596
Somnolence	47	3.1	73	4.6	207	7.6	326	9.1	627	11.8	1,068	11.5	2,354	10.9	59	4.0	4,761
Rash	427	28.6	326	20.6	352	13.0	353	9.9	472	8.9	597	6.4	1,055	4.9	65	4.4	3,647
Nausea	13	0.9	50	3.2	96	3.5	167	4.7	282	5.3	561	6.0	1,172	5.4	38	2.6	2,379
Pruritus	145	9.7	126	8.0	158	5.8	186	5.2	296	5.6	408	4.4	732	3.4	17	1.2	2,068
Vomiting	28	1.9	31	2.0	76	2.8	89	2.5	125	2.4	296	3.2	763	3.5	27	1.8	1,435
Urticaria	97	6.5	100	6.3	130	4.8	160	4.5	211	4.0	266	2.9	414	1.9	16	1.1	1,394
Constipation	12	0.8	19	1.2	51	1.9	108	3.0	162	3.0	300	3.2	728	3.4	11	0.7	1,391
Mouth Dry	-	0.0	3	0.2	32	1.2	67	1.9	97	1.8	268	2.9	695	3.2	17	1.2	1,179
Dyspepsia	9	0.6	13	0.8	43	1.6	54	1.5	119	2.2	259	2.8	539	2.5	13	0.9	1,049
Headache	1	0.1	21	1.3	64	2.4	95	2.7	141	2.7	187	2.0	393	1.8	21	1.4	923
Hepatic Enzymes Increased	53	3.5	28	1.8	51	1.9	42	1.2	91	1.7	134	1.4	228	1.1	154	10.5	781
Asthenia	8	0.5	15	0.9	9	0.3	36	1.0	44	0.8	120	1.3	292	1.4	5	0.3	529
Tremor	4	0.3	12	0.8	47	1.7	48	1.3	52	1.0	69	0.7	280	1.3	12	0.8	524
Paraesthesia	1	0.1	13	0.8	43	1.6	81	2.3	84	1.6	94	1.0	143	0.7	21	1.4	480
Fever	32	2.1	49	3.1	55	2.0	57	1.6	48	0.9	83	0.9	148	0.7	5	0.3	477
Oedema Generalised	6	0.4	1	0.1	2	0.1	18	0.5	42	0.8	110	1.2	293	1.4	4	0.3	476
Face Oedema	2	0.1	2	0.1	12	0.4	13	0.4	35	0.7	103	1.1	301	1.4	3	0.2	471
Oedema	5	0.3	1	0.1	6	0.2	15	0.4	39	0.7	92	1.0	275	1.3	6	0.4	439
Insomnia	8	0.5	3	0.2	20	0.7	41	1.1	48	0.9	83	0.9	201	0.9	32	2.2	436
Weight Increase	8	0.5	13	0.8	46	1.7	41	1.1	46	0.9	93	1.0	151	0.7	36	2.5	434
Thrombocytopenia	42	2.8	33	2.1	15	0.6	8	0.2	26	0.5	35	0.4	184	0.9	11	0.7	354
Diarrhoea	19	1.3	9	0.6	19	0.7	23	0.6	28	0.5	52	0.6	195	0.9	4	0.3	349
Vision Abnormal	1	0.1	10	0.6	23	0.8	42	1.2	25	0.5	65	0.7	146	0.7	16	1.1	328
Malaise	2	0.1	4	0.3	11	0.4	11	0.3	47	0.9	93	1.0	146	0.7	6	0.4	320
Oedema Peripheral	1	0.1	-	0.0	4	0.1	7	0.2	17	0.3	39	0.4	230	1.1	9	0.6	307
Leucopenia	50	3.3	15	0.9	30	1.1	20	0.6	28	0.5	59	0.6	85	0.4	8	0.5	295
Anorexia	6	0.4	26	1.6	27	1.0	13	0.4	38	0.7	44	0.5	118	0.5	15	1.0	287
Abdominal Pain	6	0.4	5	0.3	18	0.7	27	0.8	30	0.6	55	0.6	118	0.5	6	0.4	265
Apathy	1	0.1	4	0.3	5	0.2	25	0.7	24	0.5	51	0.5	139	0.6	7	0.5	256
Others	427	28.6	494	31.3	771	28.4	906	25.3	1,124	21.1	1,838	19.8	4,105	19.1	668	45.5	10,333

ADR: adverse drug reaction

Table S3. The most commonly reported adverse drug reactions according to sex

ADR	Total		Male		Female		Unknown	
	N	%	N	%	N	%	N	%
Total	46,963	100.0	16,349	100.0	29,454	100.0	1,160	100.0
Dizziness	8,596	18.3	2,315	14.2	6,137	20.8	144	12.4
Somnolence	4,761	10.1	1,607	9.8	3,089	10.5	65	5.6
Rash	3,647	7.8	1,605	9.8	1,992	6.8	50	4.3
Nausea	2,379	5.1	537	3.3	1,803	6.1	39	3.4
Pruritus	2,068	4.4	818	5.0	1,228	4.2	22	1.9
Vomiting	1,435	3.1	312	1.9	1,100	3.7	23	2.0
Urticaria	1,394	3.0	593	3.6	789	2.7	12	1.0
Constipation	1,391	3.0	596	3.6	782	2.7	13	1.1
Mouth Dry	1,179	2.5	393	2.4	772	2.6	14	1.2
Dyspepsia	1,049	2.2	324	2.0	713	2.4	12	1.0
Headache	923	2.0	282	1.7	628	2.1	13	1.1
Hepatic Enzymes Increased	781	1.7	459	2.8	315	1.1	7	0.6
Asthenia	529	1.1	154	0.9	361	1.2	14	1.2
Tremor	524	1.1	188	1.1	325	1.1	11	0.9
Paraesthesia	480	1.0	131	0.8	338	1.1	11	0.9
Fever	477	1.0	218	1.3	251	0.9	8	0.7
Oedema Generalised	476	1.0	90	0.6	384	1.3	2	0.2
Face Oedema	471	1.0	105	0.6	358	1.2	8	0.7
Oedema	439	0.9	112	0.7	321	1.1	6	0.5
Insomnia	436	0.9	130	0.8	279	0.9	27	2.3
Weight Increase	434	0.9	93	0.6	311	1.1	30	2.6
Thrombocytopenia	354	0.8	173	1.1	170	0.6	11	0.9
Diarrhoea	349	0.7	168	1.0	173	0.6	8	0.7
Vision Abnormal	328	0.7	112	0.7	204	0.7	12	1.0
Malaise	320	0.7	90	0.6	227	0.8	3	0.3
Oedema Peripheral	307	0.7	113	0.7	189	0.6	5	0.4
Leucopenia	295	0.6	141	0.9	151	0.5	3	0.3
Anorexia	287	0.6	118	0.7	148	0.5	21	1.8
Abdominal Pain	265	0.6	68	0.4	190	0.6	7	0.6
Apathy	256	0.5	61	0.4	189	0.6	6	0.5
Others	10,333	22.0	4,243	26.0	5,537	18.8	553	47.7

ADR: adverse drug reaction

Table S4. Detected signals of adverse drug reactions in sexual/reproductive disorder associated with anti-seizure medications.

Drug	ADR	Reports (n)	PRR	ROR	IC	χ^2	Signal
Reproductive disorders, male							
Clonazepam	Ejaculation Disorder	1	17.24	17.24	3.60	7.65	N
	Ejaculation Premature	1	8.62	8.62	3.19	4.49	N
Phenobarbital	Balanoposthitis	1	.	.	7.62	196.33	N
Valproate	Ejaculation Premature	1	5.31	5.31	2.54	2.33	N
	Semen Abnormal	1	.	.	3.54	10.62	N
Carbamazepine	Ejaculation Premature	1	6.87	6.87	2.88	3.34	N
Topiramate	Sexual Function Abnormal	2	4.20	4.20	2.31	3.99	N
Reproductive disorders, female							
Gabapentin	Breast Pain	3	8.09	8.09	1.56	4.66	N
Valproate	Amenorrhoea	3	7.97	7.97	2.54	10.45	Y
	Menstrual Disorder	10	5.06	5.07	2.00	22.08	Y
	Dysmenorrhoea	1	5.31	5.31	2.54	2.33	N
	Gynecological-Related Pain	1	.	.	3.54	10.62	N
Carbamazepine	Breast Pain	1	4.58	4.58	2.56	2.10	N
	Lactation Nonpuerperal	1	4.58	4.58	2.56	2.10	N
Topiramate	Amenorrhoea	2	7.56	7.56	2.90	8.13	N
	Breast Engorgement	2	37.78	37.81	3.90	23.87	N
	Breast Pain Female	1	18.89	18.90	3.73	8.47	N
	Menstrual Disorder	13	13.64	13.71	3.12	88.50	Y
	Dysmenorrhoea	2	37.78	37.81	3.90	23.87	N
	Leukorrhoea	1	18.89	18.90	3.73	8.47	N
	Menorrhagia	1	.	.	4.31	18.89	N
	Vaginitis	3	.	.	4.31	56.68	N
Foetal disorders							
Carbamazepine	Drug Exposure In Pregnancy	1	13.74	13.74	3.30	5.91	N
Neonatal and infancy disorders							
Topiramate	Psychomotor Development Impaired	11	.	.	4.31	207.85	N

ADR: Adverse drug reaction; PRR: Proportional reporting ratio; ROR: Reporting odds ration; IC: Information component, Chi: Chi-square test

Table S5. Detected signals of adverse drug reactions in skin and appendages disorders associated with anti-seizure medications.

**Drug**	**ADR**	**Reports (n)**	**PRR**	**ROR**	**IC**	χ^2
SCBs						
Carbamazepine	Angioedema	23	3.81	3.83	1.72	37.35
	Bullous Eruption	17	4.25	4.26	1.86	32.27
	Dermatitis Exfoliative	3	13.74	13.75	3.07	17.72
	Epidermal Necrolysis	14	4.27	4.29	1.88	26.81
	Erythema Multiforme	12	9.16	9.19	2.63	52.35
	Pruritus	350	2.80	3.02	1.32	351.51
	Rash	607	2.74	3.15	1.30	607.41
	Rash Erythematous	35	4.22	4.25	1.82	65.93
	Rash Maculo-Papular	19	3.35	3.36	1.59	25.18
	Skin Discolouration	4	3.05	3.06	1.68	4.52
	Skin Exfoliation	19	7.91	7.95	2.47	72.85
	Skin Reaction Localised	5	6.87	6.88	2.47	16.72
	Stevens Johnson Syndrome	73	7.83	7.99	2.43	278.32
	Urticaria	211	2.45	2.55	1.16	158.35
Lacosamide	Rash Erythematous	4	2.68	2.70	1.71	4.12
Lamotrigine	Epidermal Necrolysis	17	7.39	7.43	2.53	66.94
	Pruritus	333	3.50	3.90	1.64	522.86
	Rash	799	5.12	7.13	2.08	2243.68
	Rash Erythematous	16	2.20	2.20	1.13	9.33
	Rash Maculo-Papular	11	2.33	2.34	1.24	7.46
	Skin Discolouration	5	5.37	5.38	2.33	13.74
	Skin Exfoliation	9	3.82	3.83	1.86	15.52
	Stevens Johnson Syndrome	58	7.40	7.56	2.49	229.55
	Urticaria	328	5.62	6.33	2.18	980.98
Oxcarbazepine	Bullous Eruption	6	2.81	2.82	1.61	6.44
	Dermatitis	28	26.26	26.75	3.90	368.51
	Pruritus	183	3.00	3.29	1.51	233.35
	Rash	400	3.81	4.86	1.81	801.09
	Rash Erythematous	29	7.48	7.61	2.67	131.51
	Rash Maculo-Papular	22	9.08	9.20	2.91	122.53
	Skin Discolouration	4	6.88	6.89	2.80	16.45
	Urticaria	130	3.18	3.39	1.58	181.87
Phenytoin	Bullous Eruption	5	3.95	3.96	2.14	10.24
	Pruritus	78	2.07	2.18	1.04	43.53
	Rash	195	2.99	3.56	1.53	264.30
	Rash Erythematous	9	3.40	3.42	1.84	14.36
	Stevens Johnson Syndrome	16	4.57	4.64	2.18	41.27
Valproate	Acne	6	2.45	2.45	1.30	4.19
	Alopecia	96	11.72	11.99	2.62	449.42
	Epidermal Necrolysis	13	3.00	3.01	1.44	13.55
	Hypotrichosis	3	6.38	6.38	2.37	8.50
	Rash Maculo-Papular	16	2.10	2.10	1.01	7.70
	Skin Reaction Localised	6	7.08	7.09	2.35	18.81
Topiramate	Sweating Decreased	10	188.91	189.71	4.19	169.96
Non-SCBs						
Clonazepam	Sweating Increased	16	2.53	2.54	1.30	12.95

Drug	ADR	Reports (n)	PRR	ROR	IC	χ^2
Levitiracetam	Acne	8	4.51	4.52	1.99	16.39
	Skin Dry	3	4.51	4.51	2.16	6.15
Phenobarbital	Pruritus	23	2.21	2.34	1.19	15.73
	Rash	61	3.34	4.15	1.74	106.59
	Stevens Johnson Syndrome	5	5.01	5.09	2.55	15.71
	Urticaria	20	2.86	3.03	1.56	24.53
Pregabalin	Sweating Increased	59	2.25	2.25	0.74	21.62

ADR: Adverse drug reaction; PRR: Proportional reporting ratio; ROR: Reporting odds ration; IC: Information component, Chi: Chi-square test

Table S6. Detected signals of adverse drug reactions in psychiatric disorders associated with anti-seizure medications.

Drug	ADR	Reports (n)	PRR	ROR	IC	χ^2
SCBs						
Carbamazepine	Drug Dependence	3	10.30	10.31	2.88	14.40
Lacosamide	Anorexia	8	2.79	2.82	1.62	8.98
	Depression	9	5.65	5.74	2.57	32.64
	Emotional Lability	3	10.06	10.12	3.57	22.20
Lamotrigine	Paroniria	10	7.02	7.05	2.52	37.32
Phenytoin	Confusion	6	2.81	2.82	1.65	6.64
Topiramate	Aggressive Reaction	9	4.36	4.37	2.02	18.95
	Amnesia	43	9.34	9.49	2.74	214.81
	Anorexia	92	8.91	9.23	2.68	441.82
	Concentration Impaired	5	23.61	23.66	3.58	48.13
	Confusion	15	2.73	2.74	1.41	14.35
	Depression	31	4.40	4.45	1.95	66.36
	Depression Aggravated	3	28.34	28.37	3.73	31.65
	Emotional Lability	11	9.90	9.94	2.85	57.76
	Hallucination	8	2.22	2.23	1.22	4.82
	Insomnia	70	3.61	3.69	1.69	112.09
	Nervousness	13	3.23	3.24	1.63	17.14
	Sleep Disorder	18	2.70	2.71	1.38	16.89
	Thinking Abnormal	14	3.89	3.91	1.85	24.96
Valproate	Appetite Increased	28	3.31	3.32	1.50	34.43
	Manic Reaction	4	42.50	42.54	3.28	32.42
	Psychosomatic Disorder	3	31.87	31.90	3.22	22.43
	Sleep Disorder	24	2.13	2.13	1.00	11.95
Non-SCBs						
clonazepam	Amnesia	25	4.10	4.13	1.86	47.54
	Anxiety	17	2.57	2.58	1.31	14.24
	Apathy	39	3.10	3.13	1.50	47.23
	Confusion	17	2.87	2.89	1.45	17.84
	Delirium	51	10.34	10.53	2.79	269.79
	Depersonalization	5	4.31	4.32	2.07	10.17
	Dreaming Abnormal	4	9.85	9.86	2.93	20.25
	Hallucination	11	2.92	2.93	1.51	11.87
	Impotence	8	5.30	5.32	2.23	21.38
	Insomnia	46	2.03	2.05	0.97	21.81
	Paroniria	12	8.62	8.65	2.68	53.92
	Personality Disorder	4	3.13	3.14	1.76	4.92
	Sleep Disorder	31	4.73	4.77	2.01	71.75
	Somnolence	515	2.09	2.36	0.98	290.87
	Suicide Attempt	9	5.97	5.98	2.34	27.66
	Thinking Abnormal	14	3.55	3.56	1.72	21.29
Pregabalin	Appetite Increased	57	2.35	2.35	0.78	22.88
Levetiracetam	Aggressive Reaction	28	18.94	19.09	3.10	198.41
	Agitation	9	5.07	5.08	2.09	21.42
	Anxiety	19	2.29	2.30	1.14	11.90
	Depression	30	3.03	3.05	1.45	33.42
	Emotional Lability	8	4.51	4.52	1.99	16.39
	Euphoria	3	10.14	10.15	2.86	14.13
	Nervousness	35	8.77	8.85	2.54	146.40
	Personality Disorder	13	13.53	13.58	2.91	75.44

Drug	ADR	Reports (n)	PRR	ROR	IC	χ^2
	Psychosis	4	13.53	13.54	3.01	23.20
	Suicide Attempt	6	2.80	2.80	1.50	5.75

ADR: Adverse drug reaction; PRR: Proportional reporting ratio; ROR: Reporting odds ration; IC: Information component, Chi: Chi-square test
